# Magnetic Solid-Phase Extraction Using Fe_3_O_4_@SiO_2_ Magnetic Nanoparticles Followed by UV-Vis Spectrometry for Determination of Paraquat in Plasma and Urine Samples

**DOI:** 10.1155/2017/8704639

**Published:** 2017-07-17

**Authors:** Ou Sha, Yu Wang, Xin Yin, Xiaobing Chen, Li Chen, Shujun Wang

**Affiliations:** ^1^Analysis and Test Center of Jiangsu Marine Resources Development Research Institute, Huaihai Institute of Technology, Lianyungang, China; ^2^School of Chemistry and Chemical Engineering, Huaihai Institute of Technology, Lianyungang, China; ^3^Jiangsu Key Laboratory of Marine Pharmaceutical Compound Screening, Huaihai Institute of Technology, Lianyungang, China; ^4^The First People's Hospital of Lianyungang City, Lianyungang, China

## Abstract

A rapid and simple method was optimized and validated for the separation and quantification of paraquat, a frequently used herbicide and a leading cause of fatal poisoning worldwide, at trace levels with UV-Vis spectrophotometry in plasma and urine samples by direct magnetic solid-phase extraction. Fe_3_O_4_@SiO_2_ nanoparticles (NPs) were used as the magnetic solid-phase extraction agents and the paraquat absorbed on NPs was eluted using NaOH and ascorbic acid. Upon optimization, paraquat could be extracted and concentrated from various samples by 35-fold. The linear range, limit of detection (LOD), correlation coefficient (*R*), and relative standard deviation (RSD) could reach 15.0–400.0 *μ*g/L, 12.2 *μ*g/L, 0.9987, and 0.65% (*n* = 5, *c* = 40.0 *μ*g/L), respectively. The Fe_3_O_4_@SiO_2_ NPs could be reused up to five times. The method was successfully applied to the determination of paraquat in urine and plasma at different hemoperfusion numbers in a local hospital for the patient of paraquat poisoning. The experiment result could not only enable immediate medical intervention but also benefit patients' survival.

## 1. Introduction

Paraquat (1,1-dimethyl-4,4-bipyridinium ion, PQ) is a highly effective herbicide, widely used on various crops for the last 60 years in over 130 countries. Although its standard use does not represent a serious poisoning risk to man due to its inactivation by the natural components of the soil, the ingestion of PQ often leads to severe clinical situations [1]. There were about 4000 deaths from paraquat poisoning reported in China between 1991 and 2008 [2], and the occurrence is on the rise. With no known antidote available, treatment of paraquat mostly relies on its removal from patients' blood by hemoperfusion therapy and forced diuresis [3]. If the PQ concentrations in plasma and urine remain greater than 3 mg/L and 1 mg/L within a 24-hour period, respectively, the probability of patients' survival is very low [4–6]. Therefore, rapid and accurate detection of PQ level within plasma and urine samples will help physicians to decide on treatment strategies and improves patients' survival.

During the last few decades, a number of analytical methods have been reported for the identification and quantitation of paraquat in biological samples, which includes spectrophotometry [7, 8], gas chromatography (GC) [9], gas chromatography-mass spectrometry (GC-MS) [10, 11], high performance liquid chromatography (HPLC) [12, 13], high performance liquid chromatography coupled with mass spectrometry (HPLC-MS) [14], resonance light scattering technique (RLST) [15], voltammetry [16], and derivative spectroscopy [17]. Most of these methods often require expensive devices and professional operators, are associated with high cost, and thus are limited in clinical use. The UV spectrometry method, albeit generally accurate, relatively cheap, and simple to operate, is not well suited for PQ detection mainly due to matrix effects and the low concentration of PQ in biological samples. These drawbacks severely limit the availability of a diagnostic technology for detecting PQ poisoning, especially in remote and poor areas where the occurrence is particularly high. Therefore, developing an effective and inexpensive method for detecting PQ from different biological samples is of urgent importance for lowering fatality from PQ poisoning in clinic.

Recently, magnetic solid-phase extraction (MSPE) is increasingly used for sample preparation, as an adsorbent for the removal and/or preconcentration of many kinds of inorganic or organic pollutants [18, 19]. In MSPE procedures, magnetic adsorbents can be easily magnetized and gathered together in the presence of an external magnetic field and redispersed immediately once the magnetic field is removed, which greatly simplifies the SPE procedure and enhances the extraction efficiency. Most MSPE sorbents contain Fe_3_O_4_ nanoparticles (NPs) with modifications on surface chemical functional groups to improve specific binding to the target analytes and thus enable concentrations. Numerous organic and inorganic polymers have been used to modify Fe_3_O_4_ NPs [20, 21].

In the present study, we proposed, for the first time, to combine Fe_3_O_4_@SiO_2_ NPs solid-phase extraction with UV-Vis for the absorption and determination of PQ in the plasma and urine samples free from any proteins and eluted PQ by sodium hydroxide-ascorbic acid. The free radical, the reduction product of PQ following the elution, is used for quantitative detection of PQ by UV-Vis spectrophotometry. Compared with the other reported methods in literature, the present method requires a simple and fast pretreatment of samples and presented a low limit of detection. The Fe_3_O_4_@SiO_2_ NPs could be reused for five times. This method has been successfully applied to detect PQ in real plasma and urine sample, demonstrating high sensitivity that allows for fast assessment of patient outcome based on routine clinical parameters following the history of paraquat ingestion and thus facilitates the decision on treatment strategies.

## 2. Experimental

### 2.1. Reagents

All reagents were of analytical grade or higher in purity and purchased from Sinopharm Chemical Reagent Co., Ltd. (Shanghai, China), unless otherwise specified. Milli-Q water (Millipore, Bedford, MA, USA) was used throughout the study. Britton-Robinson (B-R) buffer solutions were prepared by blending the mixed acid (0.04 mol/L H_3_PO_4_, HAc and H_3_BO_3_) with 0.2 mol/L NaOH in proportion. PQ was obtained from the Chinese National Institute for the Control of Pharmaceutical and Biological Products (Beijing, China) without further treatment. A 1000 ng/mL of paraquat stock solution was prepared by dissolution of the dichloride salt in water. Working solutions were prepared by dilution of the stock solution with water. Sodium dithionite was unstable in the presence of moisture and kept in a desiccator.

### 2.2. Apparatus

All spectra measurements were carried out using a model UV-2501 spectrophotometer (Shimadzu Corporation, Japan) with matched quartz cells. All pH values were measured by a PHS-25B pH meter (Shanghai Precision & Scientific Instrument Co., Ltd., Shanghai, China). FT-IR spectra were measured with a Bruker Tensor 27 spectrometer (Bruker Company, Germany). Samples were pressed into KBr pellets and recorded at the frequencies from 4000 to 400 cm^−1^ with a resolution of 4 cm^−1^.

### 2.3. Preparation of Fe_3_O_4_@SiO_2_ NPs

Fe_3_O_4_ NPs were prepared by the conventional coprecipitation method [22]. First, FeCl_3_ (6.28 g) was dissolved in deionized water (150 mL) followed by addition of polyethylene glycol (30 mL, 20%, w/w) and of (NH_4_)_2_Fe(SO_4_)_2_ (7.84 g) water solution under stirring. Then ammonium hydroxide (20 mL, 26.5%, w/w) was added rapidly under vigorous stirring. The resultant solution was stirred (1000 r·min^−1^) at 80°C for 60 min. After cooling to room temperature, the obtained Fe_3_O_4_ precipitate was collected by an external magnetic field, washed with deionized water five times, and dried at 60°C for 12 h in vacuum.

Fe_3_O_4_@SiO_2_ was prepared according to the literature method [23]. Fe_3_O_4_ (1.0 g) were dissolved in 150 mL of ethanol and 30 mL of deionized water by sonication for 15 min, and then 2 mL of ammonium hydroxide and 4 mL of tetraethyl orthosilicate (TEOS) were added. The mixture was allowed to react for 6 h at 60°C under continuous stirring. The resultant product was collected by an external magnetic field, rinsed with deionized water and ethanol for six times thoroughly, and dried in vacuum to obtain Fe_3_O_4_@SiO_2_ NPs.

### 2.4. MSPE Procedure

**Figure 1:**
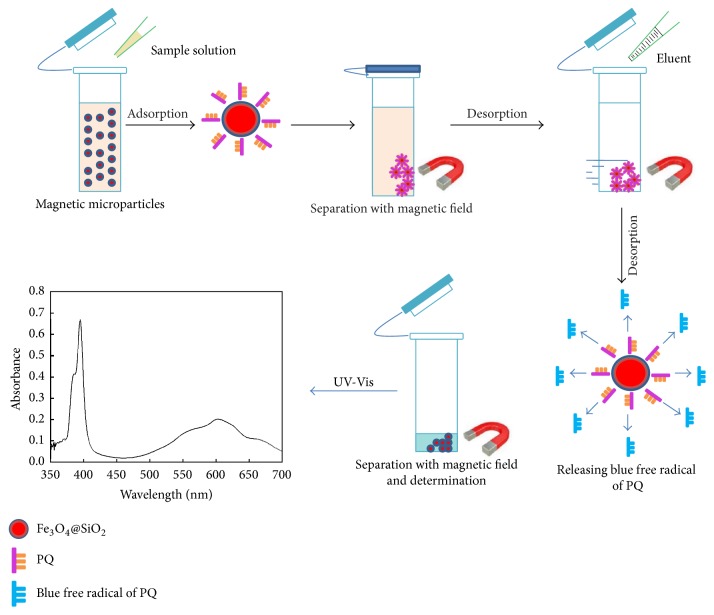
Schematic of the MSPE procedure for detecting PQ using Fe_3_O_4_@SiO_2_ NPs.

To process a plasma or urine sample, 20.0 mg of Fe_3_O_4_@SiO_2_ NPs was added to a 40 mL polyethylene tube with a cap, and the spiked plasma or urine sample was pipetted into the tube. The tube was vortexed for 10 min for extraction. Then the Fe_3_O_4_@SiO_2_ NPs were gathered and kept static for 1 min with an externally applied Nd-Fe-B permanent magnet. The Fe_3_O_4_@SiO_2_ NPs adsorbent was washed twice under vertexing (30 s/wash) in 1 mL water. Once the suspension became limpid, the supernatant was pipetted out. The isolated Fe_3_O_4_@SiO_2_ NPs were eluted with 0.2 mL of 6.0 mol/L NaOH and 0.8 mL of 50 g/L ascorbic acid to elute the preconcentrated target analytes. The eluted solution was collected and then transferred into a microcell for the detection of target analyte by UV-Vis. The whole time of the process was about 20 min. The general extraction procedure is illustrated in Figure 1.

### 2.5. Sample Preparation

This study was approved by the Ethics Committee of the Huaihai Institute of Technology (Lianyungang, China). Human plasma and urine samples were obtained from patients of PQ intoxication and healthy adults with no known record of occupational exposure to PQ (as blank) from the First People's Hospital of Lianyungang (as detailed below). Written consent was signed by all participants. Plasma and urine samples spiked with standard PQ stock solutions to indicate concentrations were used as positive controls. The venous blood was drawn from the forearm of each subject and immediately transferred to vessels containing heparin as an anticoagulant. Plasma was separated by centrifugation at 4000 rpm for 10 min and stored at 4°C.

Urine samples were collected in accordance with conventional sampling practices using 100 mL clear vials with polytetrafluoroethylene-lined screw caps. The samples were stored at −4°C in a refrigerator.

## 3. Results and Discussion

### 3.1. Characterization of the Fe_3_O_4_@SiO_2_ NPs

**Figure 2:**
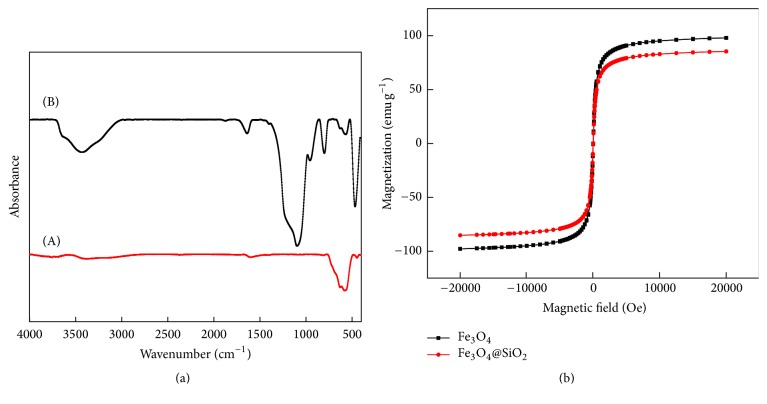
FT-IR spectra and magnetic hysteresis loops: (a) FT-IR spectra of Fe_3_O_4_ (A) and Fe_3_O_4_@SiO_2_ (B) and (b) magnetic hysteresis loops of Fe_3_O_4_ and Fe_3_O_4_@SiO_2_.

The Fe_3_O_4_ and Fe_3_O_4_@SiO_2_ MNPs were characterized using Fourier transform infrared (FT-IR) spectroscopy (Figure 2(a)) and magnetic characterization (Figure 2(b)), respectively. As shown in Figure 2(a), the peak at 580 cm^−1^ in curve (A) was assigned to Fe-O-Fe stretching vibrations and the strong peaks at 1200–1000 cm^−1^ in curve (B) corresponded to Si-O-H and Si-O-Si stretching vibrations, indicating that SiO_2_ had been successfully loaded onto the surface of Fe_3_O_4_. The magnetic hysteresis loops of the two types of MNPs were shown in Figure 2(b). It is apparent that both of Fe_3_O_4_ and Fe_3_O_4_@SiO_2_ showed superparamagnetic properties due to the core magnetite particles. Magnetic characterization using a magnetometer at 25°C indicated that the maximal saturation magnetizations of the Fe_3_O_4_ and Fe_3_O_4_@SiO_2_ were 96.3 and 84.7, respectively. Due to their superparamagnetism and large saturation magnetization, Fe_3_O_4_@SiO_2_ could be rapidly separated from the solution using a magnet.

### 3.2. Optimizations of PQ Extraction

To optimize the extraction procedure of PQ, we focused on the following factors: pH, the amount of Fe_3_O_4_@SiO_2_ MNPs, temperature, the extraction time, and the sample volume.

#### 3.2.1. pH

**Figure 3:**
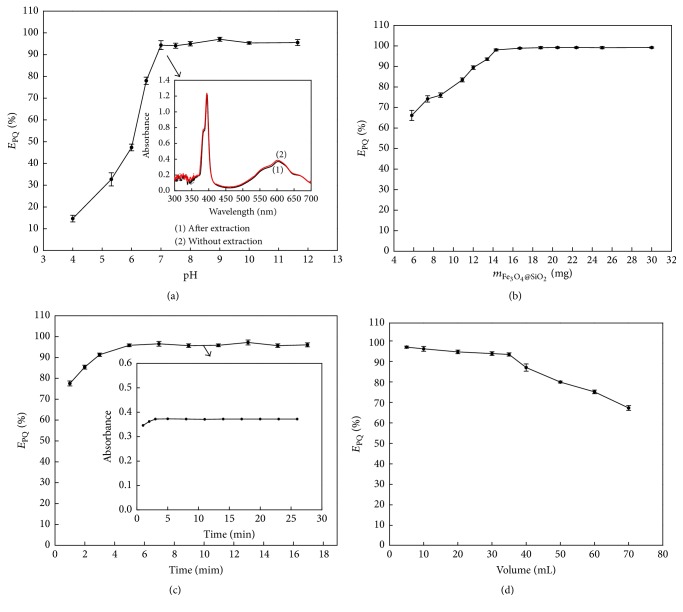
Factors affecting the extraction efficiency. The extraction efficiency (*E*_PQ_%) was determined as a function of pH (a), the amount of Fe_3_O_4_@SiO_2_ (b), the extraction time (c), and the sample volume (d).

pH is a crucial factor affecting the extraction efficiency of PQ (*E*_PQ_). In this work, the effect of pH was investigated over the range of 4.0 to 13.0. As shown in Figure 3(a), *E*_PQ_% increased with the pH and reached plateau between the pH range of 6.8–12.0. Therefore, pH 7.0 was selected for the subsequent assays.

The spectra of PQ reduced by ascorbic acid before and after the extraction were shown as curve 1 and curve 2, respectively, in Figure 3(b). No noticeable differences are found between the two spectral curves. This means that no direct chemical (bonding) interactions were involved between Fe_3_O_4_@SiO_2_ MNPs and PQ. The speciation of PQ was not changed after the magnetic solid-phase extraction.

#### 3.2.2. The Amount of Fe_3_O_4_@SiO_2_ MNPs

To assess the effect of the amount of Fe_3_O_4_@SiO_2_ MNPs on PQ extraction, we studied the Fe_3_O_4_@SiO_2_ MNPs within the range of 5.8 to 32 mg. As shown in Figure 3(b), *E*_PQ_% increased in a dose-dependent manner within the amount range of Fe_3_O_4_@SiO_2_ MNPs between 5.0 mg and 15 mg and kept stable after 15 mg (Figure 3(b)). Therefore 20 mg of Fe_3_O_4_@SiO_2_ MNPs was selected for further experiments.

#### 3.2.3. The Extraction Time

To evaluate the effect of extraction time on *E*_PQ_%, different extraction times ranging from 1 to 17 min were tested (Figure 3(c)). *E*_PQ_% gradually increased up to 5 min and remained relatively constant thereafter. Furthermore, the absorbance of PQ in NaOH medium following the reduction by ascorbic acid remained unaltered in response to the extraction time of 5.0–17 min. Thus the extraction time of 10 min was selected for further study.

#### 3.2.4. The Sample Volume

The effect of sample volume was examined between the sample volume of 3.0–70.0 mL that contained a fixed amount of PQ. The data (Figure 3(d)) show that *E*_PQ_% decreased when the sample volume was greater than 35.0 mL. Therefore, the maximum sample volume could be set as 35.0 mL.

#### 3.2.5. Adsorption Capacity

**Figure 4:**
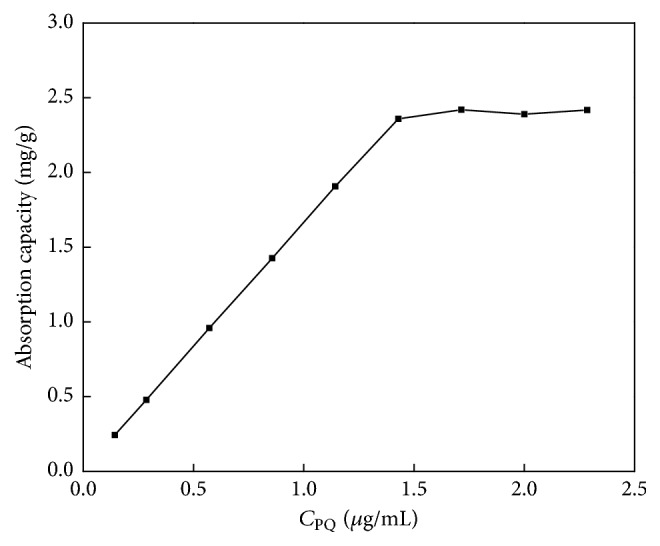
The adsorption capacity of Fe_3_O_4_@SiO_2_ MNPs.

The adsorption capacity (the maximal amount of extracted analyte from 1.0 g of extractant) is an important parameter to evaluate the efficiency. In this work, the adsorption capacity of Fe_3_O_4_@SiO_2_ MNPs for PQ was studied by varying the amount of PQ in aqueous samples before the SPE procedure. As shown in Figure 4, the adsorption capacity reached a maximum value when the concentration of PQ (*C*_PQ_) was 1.57 *μ*g·mL^−1^. Given that the amount of Fe_3_O_4_@SiO_2_ MNPs was set as 20 mg, the maximal adsorption capacity of Fe_3_O_4_@SiO_2_ MNPs was calculated as approximately 2.4 mg/g for PQ.

### 3.3. Optimization of Desorption

#### 3.3.1. The Selection and the Volume of Eluents

**Figure 5:**
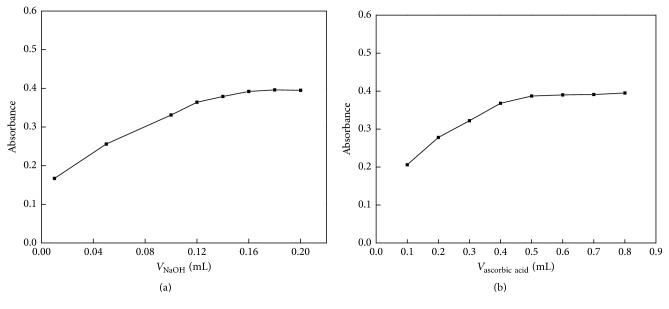
The volume effect of NaOH and ascorbic acid.

Though PQ could be absorbed by Fe_3_O_4_@SiO_2_ NPs, its reduction product, the blue free radical, could not be absorbed. Utilizing this feature, NaOH and ascorbic acid, which provided the alkaline environment and acted as reductant, respectively, were adopted as eluents in this study. The volume effect of NaOH and ascorbic acid solution on the elution efficiency of PQ was evaluated in detail. The volume of total eluents, including NaOH, ascorbic acid, and water, was fixed at 1.0 mL. The results showed that absorbance was kept stable when the volumes of 6.0 mol/L NaOH and 50 g/L ascorbic acid were higher than 0.16 mL and 0.5 mL, respectively (Figures 5(a) and 5(b)). So 0.2 mL 6 mol/L NaOH and 0.8 mL 50 g/L ascorbic acid were selected. The preconcentration factor (the quotient of volume before absorption and after elution) was 35.

#### 3.3.2. Elution Time

**Figure 6:**
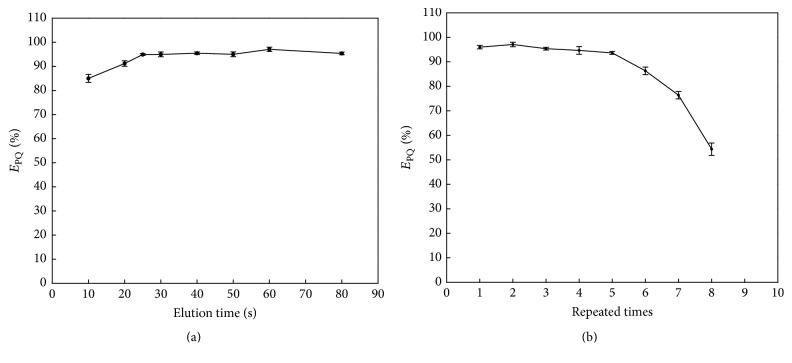
The effect of the elution time and the reusability.

The experimental results showed that the elution efficiency of NaOH and ascorbic acid increased up to 95.0% after 30 s stripping time (Figure 6(a)). Thus, 1 min was selected as the optimal elution time.

#### 3.3.3. Reusability of the Fe_3_O_4_@SiO_2_

After desorption, the Fe_3_O_4_@SiO_2_ was washed for 30 s twice by the mixture solution (1 mL 6.0 mol/L NaOH and 1 ml 50 g/L ascorbic acid) and water, respectively. Then it was dried with heating at 105°C. The extraction efficiency of the recycled Fe_3_O_4_@SiO_2_ for PQ remained above 95.0% after at least five recycles (Figure 6(b)). Thus, the Fe_3_O_4_@SiO_2_ could be used repeatedly for five times.

### 3.4. Tolerance of Contaminants

To assess the tolerance limit of this procedure, we set the detection sensitivity of PQ to 40 *μ*g/L. With a relative error of less than ±5%, the tolerance limits for various foreign ions were shown in Table 1 (tolerance ratio in mass). The results indicate that the majority of these contaminants presented no remarkable interference on the determination of PQ.

### 3.5. Analytical Performance

Under the optimal conditions, a linear calibration curve was established in the PQ concentration range of 15.0–400.0 *μ*g/L. The equation for the calibration graph is *A* (absorbance) = 0.0049 + 0.0016*C* (*μ*g/L), with a correlation coefficient of 0.9987. The limit of detection (LOD), defined as LOD = 3*σ*/*k* (where *σ* is the standard deviation of the blank sample and *k* is the slope of the calibration graph), was 12.2 *μ*g/L. The relative standard deviation was 0.65% (*n* = 5, *c* = 40.0 *μ*g/L). The preconcentration factor, defined as the quotient of the volumes before the absorption and after the elution, is 35-fold.

Table 2 offers a comparative analysis of the current method and the previously developed methods. It revealed that the cost of the combining MSPE with UV spectroscopy is relatively cheap for the common instrument and reagent. It is also rapid and easy to operate and has the same sensitivity, as compared to other reported methods.

To examine the performance of this proposed method, we obtained plasma and urine samples from either PQ patients or healthy controls from the First People's Hospital of Lianyungang City. In order to assess the accuracy of the proposed method, recovery studies were also performed on the corresponding samples by determining known amounts of PQ added to the samples at above-mentioned concentration levels (Table 3). Table 3 shows that the recoveries of the PQ ranged from 92.9% to 105.2%.

### 3.6. Clinical Application

This proposed method was applied to the emergency case of paraquat poisoning. A 28-year-old woman was admitted to the local hospital department after intentionally ingesting some of 20% paraquat solution for 60 minutes. Plasma and urine samples were collected before and after the hemoperfusion therapy and tested for the levels of paraquat on admission. The interval time of hemoperfusion was set as 8 hours. The plasma concentrations of paraquat, detected by this proposed method and HPLC method [26], were reduced with the increased number of the hemoperfusion therapies (Table 4). Applying the paired *t*-test using the comparative method and the proposed method, the calculated *t* values were smaller than the critical value (2.776, *α* = 0.05).

These results demonstrate the applicability of the combining MSPE with UV spectroscopy for the determination of paraquat in human plasma and urine samples.

## 4. Conclusion

Fe_3_O_4_@SiO_2_ MNPs were successfully prepared and applied as magnetic adsorbents to preconcentrate and separate PQ. Featuring the rapid collection of Fe_3_O_4_@SiO_2_ MNPs from the sample using an external magnetic field, easy elution with more environmental solvent, excellent enrichment in low time, and good precision and high accuracy, this extraction procedure proved to be a useful and convenient method for quantitative evaluation of paraquat in biological samples. Compared with other reported methods, this technique is simple, inexpensive, and fast. Through this study, the optimal conditions for PQ detection are established and validated using the plasma and urine samples from human patients suffering PQ poisoning and from healthy adults through the recovery experiment. The results indicated that the developed MSPE-UV-Vis method is suitable for detecting PQ poisoning in clinic. Early detection and quantification of PQ will no doubt benefit patient treatment and survival. We are investigating the application potential of this novel method in clinical studies and evaluating its significance on improving patient outcomes.

## Figures and Tables

**Table 1:** Effect of contaminants for detecting 40 *μ*g/L PQ.

Contaminants	Tolerance limits
K^+^, Na^+^, Zn^2+^	1000
Chlorophyll, lutein	600
Cd^2+^, Mg^2+^, PO_4_^3−^	500
2,4-D, 2,4,5-T, DDT, Kelthane, lindane	200
Cu^2+^, Ni^2+^, Ca^2+^, Fe^2+^, Mn^2+^	100
Al^3+^	40
Fe^3+^, Pb^2+^	20
Aquacide	5

DDT: dichlorodiphenyltrichloroethane.

**Table 2:** Comparison of the MSPE + UV method with other analytical techniques for extraction and determination of PQ.

Sample	Extraction and detection technique	Extract method and extractant	Linear range (*μ*g/L)	Limits of detection (*μ*g/L)	Ref.
Urine	UV-Vis	—	1000–1500	—	[8]
Human blood	UV-Vis	LLE dichloromethane	84.1–841	89.1	[24]
Human blood and urine	GC	—	1 × 10^3^–7 × 10^4^	—	[9]
Plasma and urine	GC-MS	SPE C_18_ cartridge	100–50000	50	[10]
Urine	GC-MS	HS-SPME fiber	10–1000	0.1	[11]
Plasma	HPLC-UV	—	2 × 10^5^–5 × 10^8^	—	[12]
Whole blood	HPLC-UV	—	300–30000	26	[25]
Human plasma	HPLC- UV	SPE Xtimate C18 column	20–1 × 10^4^	10	[26]
Urine	HPLC-MS	DSPE Carbon nanotube	3.75–375.0	0.94	[14]
Plasma and urine	UV-Vis	SPE SiO_2_@Fe_3_O_4_	15–400	4.7	This work

HS-SPME: headspace solid phase microextraction; DSPE: dispersive solid phase extraction.

**Table 3:** Determination of PQ in different plasma and urine samples (*n* = 3).

Sample	PQ content in sample	Added (*μ*g/L)	Found (*μ*g/L)	Recovery/%
Plasma from healthy adults	ND (3.0 mL plasma)	0	ND	—
		40.0	38.5 ± 1.7	96.2
		80.0	78.1 ± 0.8	97.6
Plasma from PQ patients	31.50 ng/mL (3.0 mL plasma)	0	9.5 ± 0.2	—
		5	14.6 ± 0.9	102.4
		10	18.8 ± 1.4	93.6
Urine from healthy adults	ND (100.0 mL)	0	ND	—
		100.0	95.8 ± 1.2	95.8
		200.0	185.8 ± 4.1	92.9
Urine from PQ patients	78.92 ng/mL (50.0 mL)	0	39.5 ± 1.4	—
		20.0	60.2 ± 3.0	103.5
		40.0	77.4 ± 1.8	94.8

ND, nondetectable.

**Table 4:** Determination of PQ in plasma and urine sample with different hemoperfusion numbers by the proposed method and HPLC (*n* = 3).

Number of HP	Plasma/(×10^3^ *μ*g/L)		Urine/(×10^3^ *μ*g/L)	
	The proposed method	HPLC	The proposed method	HPLC
0	6.84 ± 0.04	6.89 ± 0.04	8.54 ± 0.08	8.63 ± 0.07
1	3.32 ± 0.09	3.44 ± 0.10	1.26 ± 0.12	1.42 ± 0.13
2	1.05 ± 0.12	1.19 ± 0.11	0.24 ± 0.05	0.20 ± 0.05
3	0.34 ± 0.09	0.45 ± 0.09	ND	ND
